# Hierarchical Architecturing for Layered Thermoelectric Sulfides and Chalcogenides

**DOI:** 10.3390/ma8031124

**Published:** 2015-03-16

**Authors:** Priyanka Jood, Michihiro Ohta

**Affiliations:** Energy Technology Research Institute, National Institute of Advanced Industrial Science and Technology (AIST), Tsukuba, Ibaraki 305-8568, Japan; E-Mail: p.jood@aist.go.jp

**Keywords:** thermoelectrics, CS_2_ sulfurization, thermoelectric sulfides, misfit layered chalcogenides, homologous chalcogenides, accordion-like layered chalcogenides, thermoelectric minerals, intercalation, crystal-structural evolution, low-energy atomic vibration, stacking fault, oriented texture

## Abstract

Sulfides are promising candidates for environment-friendly and cost-effective thermoelectric materials. In this article, we review the recent progress in all-length-scale hierarchical architecturing for sulfides and chalcogenides, highlighting the key strategies used to enhance their thermoelectric performance. We primarily focus on TiS_2_-based layered sulfides, misfit layered sulfides, homologous chalcogenides, accordion-like layered Sn chalcogenides, and thermoelectric minerals. CS_2_ sulfurization is an appropriate method for preparing sulfide thermoelectric materials. At the atomic scale, the intercalation of guest atoms/layers into host crystal layers, crystal-structural evolution enabled by the homologous series, and low-energy atomic vibration effectively scatter phonons, resulting in a reduced lattice thermal conductivity. At the nanoscale, stacking faults further reduce the lattice thermal conductivity. At the microscale, the highly oriented microtexture allows high carrier mobility in the in-plane direction, leading to a high thermoelectric power factor.

## 1. Introduction

The growing demand for energy throughout the world is causing an energy crisis and aggravating environmental burden. However, more than 50% of the primary energy supplied is not utilized and is wasted in the form of heat. Solid-state devices based on thermoelectrics can directly convert the waste heat generated from various sources into useful electrical energy and can thus provide a new approach to improving energy management and sustainability while reducing greenhouse-gas emissions [1,2,3,4,5]. Currently, thermoelectric devices are found only in niche applications because thermoelectric materials show low efficiency and mainly consist of toxic and scarce (expensive) elements. Therefore, extensive efforts have been devoted to develop high-efficiency, environment-friendly, and cost-effective thermoelectric materials for large-scale practical applications, such as waste-heat recovery in vehicles and industries.

The thermoelectric figure of merit (*ZT*) expresses the efficiency of thermoelectric materials and is defined as *ZT* = (*S*^2^/ρκ_total_)*T*, where *S*, ρ, κ_total_, and *T* are the Seebeck coefficient, electrical resistivity, total thermal conductivity, and absolute temperature, respectively. An increase in the quantity *S*^2^/ρ, which is known as the thermoelectric power factor, leads to an increase in the electrical performance. The *S*^2^/ρ is typically optimized by tuning the carrier concentration of materials. The κ_total_ is the sum of two parts: the charge carriers transporting heat (*i.e.*, electronic thermal conductivity, κ_el_) and phonons traveling through the lattice (*i.e.*, lattice thermal conductivity, κ_lat_). Therefore, κ_total_ = κ_el_ + κ_lat_. The former is directly related to ρ and can be estimated using the Wiedemann-Franz law: κ_el_ = *LT*/ρ, where *L* is the Lorenz number. Therefore, one way to reduce the κ_total_ is to minimize κ_lat_, which is a carrier-independent parameter. In order to increase *S*^2^/ρ and reduce κ_lat_, thermoelectric materials have been strategically developed with the phonon glass-electron crystal (PGEC) concept proposed by Slack [6]. A PGEC system consists of an electron crystal region and a phonon glass region. The electron crystal region allows carrier transmission, resulting in high carrier mobility and high *S*^2^/ρ. The phonon glass region effectively scatters phonons, resulting in low *κ*_lat_. The PGEC concept has been realized using three methods: nanoblock integration, a nano- and meso-structuring/panoscopic approach, and rattling/low-energy atomic vibration.

Layered cobaltite oxides such as Na*_x_*CoO and Ca–Co–O have been demonstrated to be promising high-temperature thermoelectric materials [7,8,9,10,11,12,13]. An important advantage of thermoelectric oxides is their high chemical stability in air over a wide temperature range of 300–1200 K. The high *ZT* of these oxides originates from the integration of physical properties of the metallic electron-crystal nanoblock layer and disordered phonon-glass nanoblock layer. While the metallic Co–O nanoblock layers provide a high *S*^2^/ρ, the disordered Na and Ca–Co–O nanoblock layers scatter phonons and reduce κ_lat_ [10,11,12,13]. The impact of layered oxides has generated considerable interest in layered compounds, particularly layered sulfides and chalcogenides. The covalency generally increases when moving from oxides to sulfides and chalcogenides; therefore, the layered sulfides and chalcogenides show lower ρ and higher *S*^2^/ρ [14].

PbTe-based materials represent one of the most successful examples of nano- and meso-structuring [15,16,17,18,19,20,21]. The insertion of endotaxial nanostructures in PbTe bulk materials causes the effective scattering of short-mean-free-path phonons without affecting the carrier mobility. Moreover, long-mean-free-path phonons are scattered at grain boundaries by controlling and fine-tuning the mesoscale architecture of nanostructured materials. As a result, the all-length-scale hierarchical architecture (panoscopic approach) in PbTe bulk thermoelectric materials causes a significant reduction in *κ*_lat_ and dramatically increases *ZT* to an exceptionally high value of 2.2 at 915 K [17].

Skutterudites and clathrates are highly promising for practical thermoelectric devices operating in the intermediate temperature range of 573–973 K [22,23,24,25,26,27,28]. These systems contain large cages of host atoms in their crystal structures. Filling the large cages with guest atoms reduces κ_lat_ because the guest atoms rattle at low energy inside the cages and effectively scatter phonons. A very high *ZT* of 1.9 at 835 K was achieved in the skutterudite Sr_0.09_Ba_0.11_Yb_0.05_Co_4_Sb_12_ [27], and a conversion efficiency of 7% was demonstrated in a skutterudite-based thermoelectric module [28].

**Figure 1:**
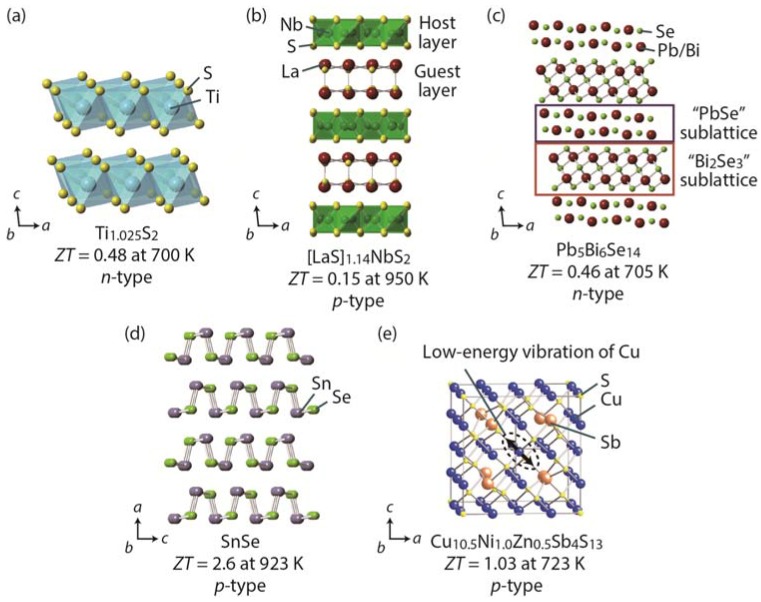
Crystal structures of the thermoelectric sulfides and chalcogenides addressed in this article: (**a**) TiS_2_ [29,30]; (**b**) [LaS]_1.14_NbS_2_ [31,32]; (**c**) Pb_5_Bi_6_Se_14_ [33,34]; (**d**) SnSe [35]; and (**e**) Cu_12_Sb_4_S_13_ [36,37]. Sizes of atoms in this figure are arbitrary.

This review discusses recent progress in an intriguing new class of thermoelectric materials, layered chalcogenides. Among the layered chalcogenides, we primarily focus on the sulfide systems because sulfur is an environment-friendly and cost-effective element. Figure 1 shows the crystal structures of the thermoelectric sulfides and chalcogenides addressed in this article, including layered sulfide TiS_2_, misfit layered sulfide [LaS]_1.14_NbS_2_, a member of cannizzarite homologous series Pb_5_Bi_6_Se_14_, accordion-like layered selenide SnSe, and thermoelectric mineral Cu_12_Sb_4_S_13_. The nanoblock integration, nano- and meso-structuring/panoscopic approach, and rattling/low-energy atomic vibration to layered sulfides and chalcogenides are important guidance for *ZT* enhancement through all-length-scale hierarchical architecture.

## 2. CS_2_ Sulfurization and Pressurized Sintering

We first discuss the synthesis and processing of thermoelectric sulfides, which drastically affects their properties. In the case of TiS_2_-based layered sulfides, a single crystal can be grown using the chemical-vapor-transport method with I_2_ as the transport agent [38,39,40,41]. Polycrystalline TiS_2_-based layered sulfides have been commonly prepared by melting stoichiometric amounts of the constituent elements in evacuated and sealed quartz tubes at 905–1273 K, followed by pressurized sintering at 873–1173 K [30,42,43,44,45,46,47]. However, these methods are difficult to apply because of the large difference in vapor pressure between metals and sulfur; consequently, there is less motivation for researchers in the thermoelectric community to investigate thermoelectric sulfides further. In this review, we focused on CS_2_ sulfurization, which is a low-temperature preparation method for thermoelectric sulfides [29,48,49,50,51,52,53].

**Figure 2:**
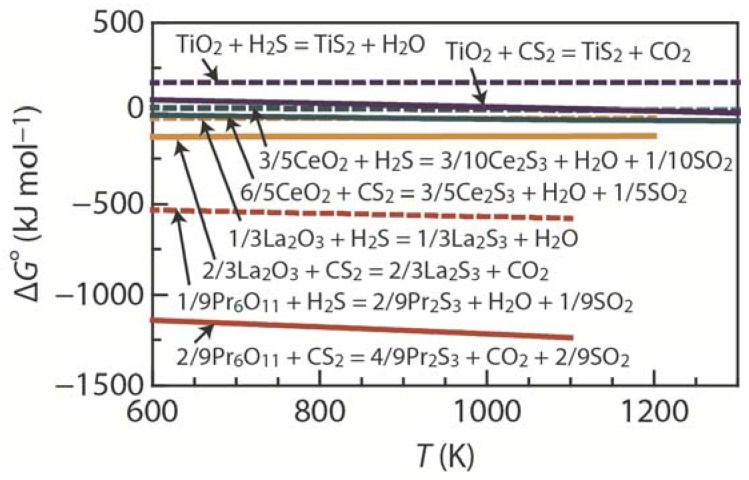
Temperature dependence of the standard free-energy change (Δ*G*°) for the sulfurization of TiO_2_ [29], La_2_O_3_, CeO_2_ [51], and Pr_6_O_11_ [52] with CS_2_ and H_2_S.

The temperature dependence of the standard free-energy change (Δ*G*°) for the sulfurization of TiO_2_, La_2_O_3_, CeO_2_, and Pr_6_O_11_ with CS_2_ and H_2_S gases, calculated from thermodynamic data [54,55], is shown in Figure 2 [29,51,52]. The value of Δ*G*° for CS_2_ sulfurization is lower than that for H_2_S sulfurization in the temperature range of 600–1300 K. For example, the values of Δ*G*° at 1100 K for the sulfurization of La_2_O_3_ with CS_2_ and H_2_S are −126 and −26 kJ·mol^−1^, respectively, which implies that CS_2_ gas is a powerful sulfurizing agent for oxides, allowing the low-temperature formation of sulfides.

**Figure 3:**
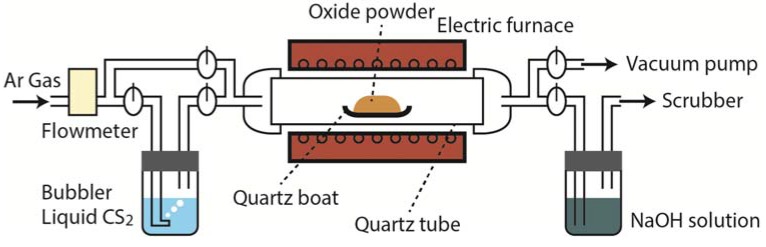
Schematic diagram of the experimental apparatus used for CS_2_ sulfurization. The apparatus was referenced previously in a report by Hirai *et al.* [51].

A schematic diagram of the experimental apparatus used for CS_2_ sulfurization is shown in Figure 3. The apparatus was referenced previously in a report by Hirai *et al.* [51]. The starting oxide powders were placed in quartz boats and set in a quartz reaction tube. The quartz tube was evacuated and purged with Ar gas. The powders were heated to 573 K under Ar gas flow. A mixture of CS_2_ and Ar gases was introduced into the quartz tube as soon as the powders reached 573 K. The CS_2_ gas was obtained by bubbling Ar carrier gas through CS_2_ liquid. Single phases of TiS_2_, [LaS]_1.20_CrS_2_, and [LaS]_1.14_NbS_2_ were successfully prepared through CS_2_ sulfurization at 1073 K for 4–12 h [29,32].

**Figure 4:**
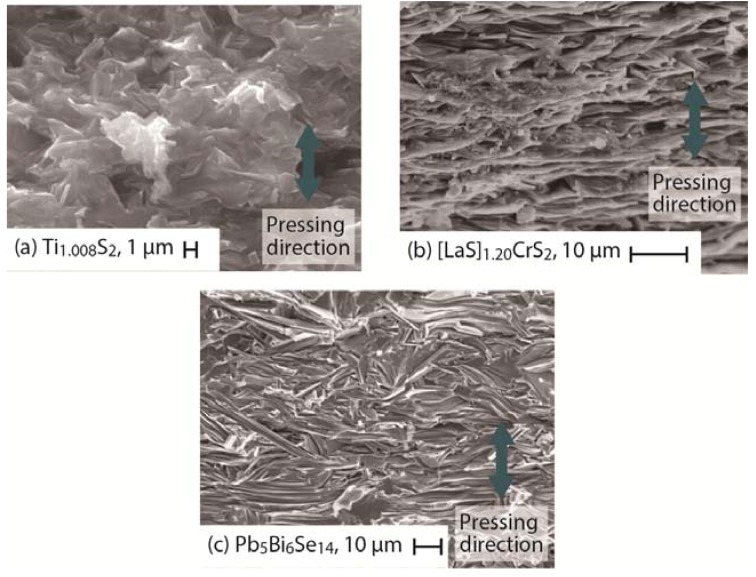
Scanning electron micrographs of the fractured section of sintered compacts of (**a**) Ti_1.008_S_2_ [29]; (**b**) [LaS]_1.20_CrS_2_ [32]; and (**c**) Pb_5_Bi_6_Se_14_ [34].

Powders of layered thermoelectric sulfides and chalcogenides were sintered under uniaxial pressure to obtain high-density compacts. Figure 4 shows scanning electron microscopy (SEM) micrographs of the fractured section of sintered compacts of Ti_1.008_S_2_ [29], [LaS]_1.20_CrS_2_ [32], and Pb_5_Bi_6_Se_14_ [34] that were fractured parallel to the pressure direction. The micrographs reveal a dense structure and well-organized microtexture, indicating that the pressurized sintering promotes densification and grain orientation. The XRD patterns show that the crystalline *c*-axis is preferentially oriented along the pressing (out-of-plane) direction [29,32,34]. A centrifugal heating technique also provides the dense samples with highly oriented microtexture [56]. The highly oriented microtextures allow high carrier mobility in the in-plane direction, leading to a high thermoelectric power factor.

## 3. TiS_2_-Based Layered Sulfides

**Figure 5:**
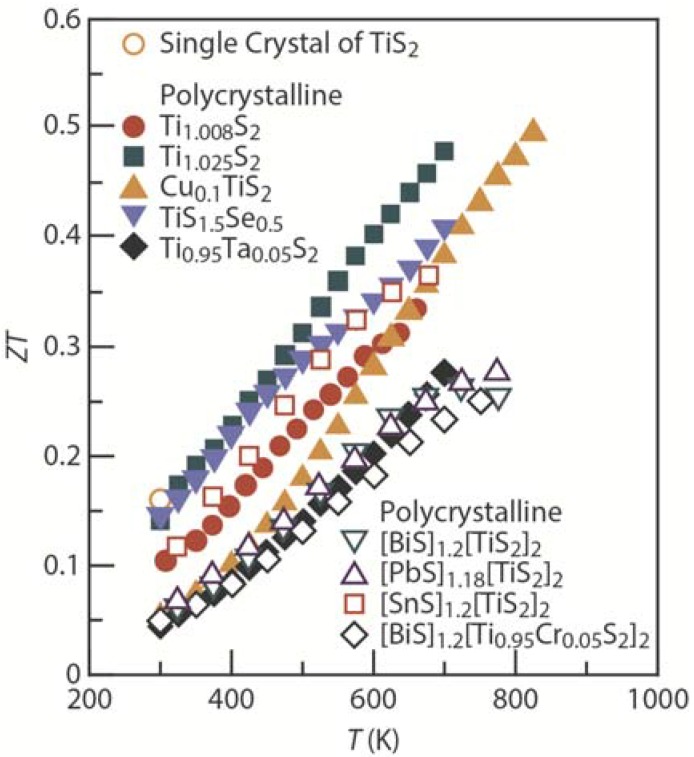
Current state-of-the-art TiS_2_-based layered sulfides. Temperature dependence of the thermoelectric figure of merit (*ZT*) in the in-plane (*ab*-plane) direction for single-crystal TiS_2_ [40] and polycrystalline Ti_1.008_S_2_ [29], Ti_1.025_S_2_ [30], Cu_0.1_TiS_2_ [44], TiS_1.5_Se_0.5_ [45], Ti_0.95_Ta_0.05_S_2_ [46], [BiS]_1.2_[TiS_2_]_2_ [42], [PbS]_1.18_[TiS_2_]_2_ [42], [SnS]_1.2_[TiS_2_]_2_ [43], and [BiS]_1.2_[Ti_0.95_Cr_0.05_S_2_]_2_ [66].

Because TiS_2_-based layered sulfides are primarily composed of earth-abundant, low-toxicity, and light elements, they will pave the way for environment-friendly, cost-effective, and lightweight thermoelectric devices [29,30,39,40,41,42,43,44,45,46,47,56,57,58,59,60,61,62,63,64,65,66]. The two-dimensional crystal structure of these systems is a great example in which the thermoelectric performance can be improved through nanoblock integration and hierarchical architecturing. The recently developed TiS_2_-based sulfides with high thermoelectric figure of merit (*ZT*) are summarized in Figure 5. In 2001, Imai *et al.* [40] demonstrated a high power factor (*S*^2^/ρ) of ~3710 μW·K^−2^·m^−1^ at 300 K in a single crystal of near-stoichiometric TiS_2_, which is comparable to that of commercially available thermoelectric materials such as Bi_2_Te_3_. However, the single crystal shows a high lattice thermal conductivity κ_lat_ of ~6.35 W·K^−1^·m^−1^, which limits the *ZT* to ~0.16 at 300 K (Table 1). Recently, the intercalation of guest atoms and guest layers into TiS_2_ layers was found to reduce the κ_lat_ of these systems, similar to layered cobaltite oxides [7,8,9,10,11,12,13]. For example, Cu intercalation reduces κ_lat_ and enhances *ZT* to ~0.5 at 823 K [44].

**Table 1:** Seebeck coefficient (*S*), electrical resistivity (ρ), carrier mobility (μ), power factor (*S*^2^/ρ), lattice thermal conductivity (κ_lat_), and thermoelectric figure of merit (*ZT*) at room temperature in the in-plane (*ab*-plane) and out-of-plane (*c*-axis) directions for a single crystal of nearly stoichiometric TiS_2_ [40] and polycrystalline Ti_1.008_S_2_ [29].

Sample	Direction	*S* (μV·K^−^^1^)	ρ (μΩ·m)	μ (cm^2^·V^−^^1^·s^−^^1^)	*S*^2^/ρ (μW·K^−2^·m^−1^)	κ_lat_ (W·K^−1^·m^−1^)	*ZT*
Single crystal	In-plane	−251	17	15	3710	6.35	0.16
Single crystal	Out-of-plane	-	13,000	0.017	-	4.21	-
Polycrystalline	In-plane	−80	6.2	2.3	1030	1.5	0.12
Polycrystalline	Out-of-plane	−84	11	1.2	630	1.3	0.10

The crystal structure of TiS_2_ consists of a stacked CdI_2_-type layer along the crystalline *c*-axis and is shown in Figure 1a. The TiS_2_ phase has a wide range of chemical compositions ranging from stoichiometric to Ti-rich: Ti_1+*x*_S_2_ with 0 ≤ *x* < 0.1 [67,68,69]. Because the TiS_2_ layer is weakly stacked through van der Waals forces, the excess Ti atoms occupy the space between TiS_2_ layers. The lattice parameter *a* monotonically increases from 0.3405 nm for Ti_1.001_S_2_ to 0.3412 nm for Ti_1.093_S_2_, and *c* increases from 0.5691 nm for Ti_1.001_S_2_ to 0.5711 nm for Ti_1.093_S_2_ [70].

To interpret the thermoelectric properties of TiS_2_, we used the following formulas for approximations of electrical resistivity ρ, carrier mobility μ, κ_lat_, and Seebeck coefficient *S* [71,72,73]:

(1) $$\frac{1}{\rho }=ne\mu$$

(2) $$\mu =\frac{e\tau_{e}}{m^{*}}$$

(3) $$\kappa_{\mathrm{lat}}=\frac{1}{3}C_{v}v_{a}l_{p}$$

(4) $$S=\frac{8\pi^{2}k_{B}^{2}}{3eh^{2}}m^{*}T{\left(\frac{\pi }{3n}\right)}^{2/3}$$

where *n*, *e*, *τ*_e_, m*, *C*_v_, *v*_a_, *l*_p_, *k*_B_, and *h* are the carrier concentration, electronic charge, relaxation time of charge carriers, effective mass, heat capacity at constant pressure, average sound velocity, mean free path of phonons, Boltzmann constant, and Planck’s constant, respectively. These relationships are derived from the parabolic band model and energy-independent scattering time for metals and degenerate semiconductors.

The layered crystal structure results in highly anisotropic electrical and thermal transport properties [29,40,43]. Table 1 lists *S*, ρ, μ, *S*^2^/ρ, κ_lat_, and *ZT* at room temperature in the in-plane (*ab*-plane) and out-of-plane (*c*-axis) directions for a single crystal of nearly stoichiometric TiS_2_ [40] and polycrystalline Ti_1.008_S_2_ [29]. The polycrystalline sample possesses a well-organized microtexture (Figure 4a) with the crystalline *ab*-plane preferentially oriented along the in-plane direction. The room-temperature *n* of the single crystal and polycrystalline samples are ~2.8 × 10^20^ cm^−^^3^ and ~4.5 × 10^21^ cm^−^^3^, respectively. For single crystal, the out-of-plane ρ (~13,000 μΩ·m) is nearly 750 times higher than the in-plane ρ (~17 μΩ·m), and the out-of-plane μ (~0.017 cm^2^·V^−^^1^·s^−^^1^) is significantly lower than the in-plane μ (~15 cm^2^·V^−^^1^·s^−^^1^) at 300 K. Moreover, the out-of-plane κ_lat_ (~4.21 W K^−1^·m^−1^) is slightly lower than the in-plane κ_lat_ (~6.35 W·K^−1^·m^−1^) at 300 K. The highly oriented polycrystalline sample also exhibits lower ρ, higher μ*,* and higher κ_lat_ in the in-plane direction. The high value of the out-of-plane ρ and low value of the out-of-plane κ_lat_ are principally due to the reduced relaxation time (reduced mean free path) resulting from the significant electron and phonon scatterings, respectively, at the interfaces between the TiS_2_ layers (see Equations (1)–(3)). The sign of *S* is negative for both samples, confirming *n*-type carrier transport. Unlike ρ and κ_lat_, *S* was found to be insensitive to the crystal orientation in the sintered compacts; as there is no relationship between *S* and τ in Equation (1), *S* is nearly isotropic. The in-plane *S* (~−80 μV·K^−^^1^) is nearly the same as the out-of-plane *S* (~−84 μV·K^−^^1^) at 300 K. The higher *S*^2^/ρ (~1030 μW·K^−2^·m^−1^) and higher *ZT* (~0.12) in the in-plane direction at 300 K for polycrystalline Ti_1.008_S_2_ is due to the lower in-plane ρ and insensitivity of *S* to the crystal orientation.

**Figure 6:**
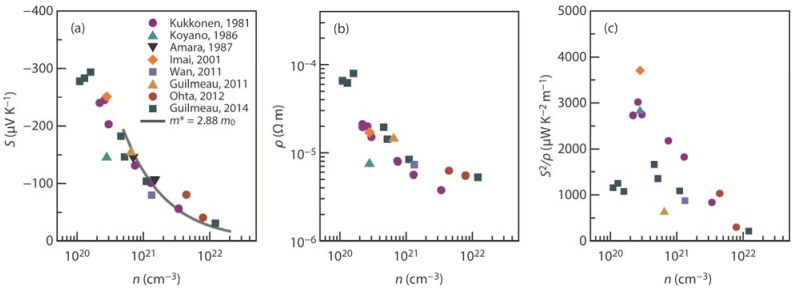
(**a**) Seebeck coefficient (*S*); (**b**) electrical resistivity (ρ); and (**c**) power factor (*S*^2^/ρ) plotted against the carrier concentration (*n*) at 300 K in the in-plane (*ab*-plane) direction for Ti_1+*x*_S_2_ [29,30,41,43,44,75,76,77]. The solid line in (**a**) represents the values calculated using Equation (4) with m^*^/*m*_0_ (*m*_0_ is the free electron mass) equal to 2.88 when *n* is greater than 5 × 10^20^ cm^−^^3^ [74,75,76].

The intercalated Ti provides *n*-type carriers to the system; therefore, *n* can be tuned to optimize *S*^2^/ρ by self-intercalation. Figure 6 shows the room-temperature *S*, ρ, and *S*^2^/ρ plotted against *n*. The solid line in Figure 6a represents the values calculated using Equation (4) with m^*^/*m*_0_ (*m*_0_ is the free electron mass) equal to 2.88 when *n* is greater than 5 × 10^20^ cm^−^^3^ [74,75,76]. The measured values fall on this calculated line. An increase in Ti content results in an increase in *n* and, hence, a decrease in *S* and ρ. The *S*^2^/ρ peaks around *n* ~ 2.8 × 10^21^ cm^−^^3^ in Ti_1+*x*_S_2_ and reaches a value of ~3700 μW·K^−2^·m^−1^.

**Figure 7:**
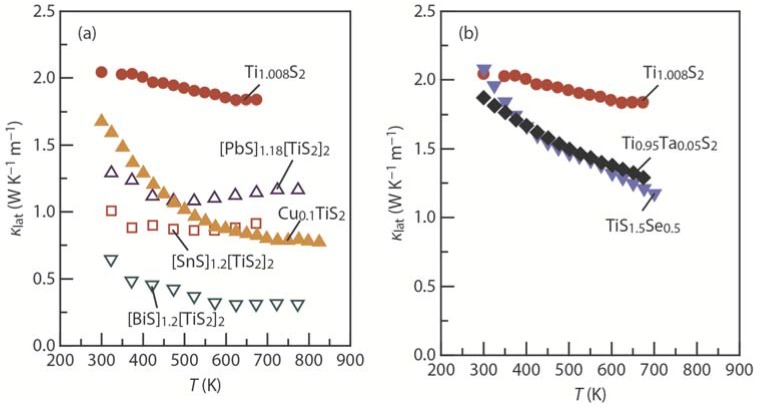
Temperature dependence of the lattice thermal conductivity (κ_lat_) in the in-plane (*ab*-plane) direction for Ti_1.008_S_2_ [29]: (**a**) intercalated systems [BiS]_1.2_[TiS_2_]_2_ [42], [PbS]_1.18_[TiS_2_]_2_ [42], [SnS]_1.2_[TiS_2_]_2_ [43], and Cu_0.1_TiS_2_ [44]; (**b**) substituted systems TiS_1.5_Se_0.5_ [45] and Ti_0.95_Ta_0.05_S_2_ [46].

One way to reduce κ_lat_ is the intercalation of guest atoms and guest layers into the host TiS_2_ layer [42,43,44,65,66]. For example, Cu intercalation reduces κ_lat_ from ~2.0 W·K^−1^·m^−1^ for TiS_2_ to ~1.7 W·K^−1^·m^−1^ for Cu_0.1_TiS_2_, and SnS intercalation dramatically reduces κ_lat_ to ~1.0 W·K^−1^·m^−1^ for [SnS]_1.2_[TiS_2_]_2_ at 300 K (Figure 7a). Substitution is another way to reduce κ_lat_ [45,46,47]; for example, the substitution of Ti by Ta slightly reduces κ_lat_ to ~1.8 W·K^−1^·m^−1^ for Ti_0.95_Ta_0.05_S_2_ at 300 K (Figure 7b).

The reduction in κ_lat_ through intercalation and substitution is limited; therefore, *ZT* is below 0.5. Future work should aim to develop new methods for a dramatic reduction in κ_lat_ to achieve *ZT* enhancement in TiS_2_-based layered sulfides. Recently, Koumoto *et al.* [64,66] have demonstrated that the stacking faults in the layers can be tuned to reduce the thermal conductivity. We believe that well-organized stacking faults can ideally reduce κ_lat_ and enhance *ZT* beyond 0.5.

## 4. Misfit Layered Sulfides [MS]_1+*m*_[TS_2_]*_n_*

TiS_2_-based layered sulfides are a part of a large family of misfit layered sulfides with a general formula [MS]_1+*m*_[TS_2_]*_n_*, where M = Sn, Pb, Sb, Bi, and rare-earth metals; T = Ti, V, Cr, Nb, and Ta; and *n* = 1, 2, 3 [78]. These compounds consist of alternating MS and TS_2_ layers, which belong to separate subgroups; therefore, these compounds lack three-dimensional periodicity. The crystal-structure analysis of rare-earth misfit compound (MS)_1+*m*_NbS_2_ has been performed using different subgroups including the (3+1)-dimensional superspace group [31,79,80], and different structural variants depending upon the intercalation layer were proposed. Miyazaki *et al.* [81] have reported a significant variation in the *c*-axis depending on the ionic radius of the rare-earth element. Previous results have shown that NbS_2_ [32,53,81,82], TaS_2_ [83,84], and TiS_2_ [42,43,65,66] sandwich structures show metallic conductivity, whereas CrS_2_ [32,85] and VS_2_ [78,85] sandwich structures show semiconducting properties. The crystal structure and physical properties of this misfit family were extensively studied in the 1990s [78]; however, not many reports on their thermoelectric properties exist to date. In recent years, the misfit layered sulfides have gained interest in the thermoelectric community for their PGEC behavior, where the intercalated NaCl-type MS layer is primarily responsible for the disorder (reduction in κ_lat_) and the CdI_2_-type TS_2_ layer behaves as a charge-carrier pathway [86,87]. The entire crystal structure is stabilized through charge transfer from the MS layer to the TS_2_ layer [78,88]. Through the concept of bond valences, it is established that the interlayer La–S bond valences are much larger than the interlayer M-S bond valences with M = Sn or Bi [88,89,90], which makes the LnS-based misfit layered compounds (Ln = rare-earth metals) much more mechanically hard (due to strong interlayer bonding) than the SnS-, PbS-, BiS-, and SbS-based systems [88].

Thus far, only [MS]_1+*m*_NbS_2_ with M = rare-earth elements [32,53,81]; [LaS]_1.2_CrS_2_ [32]; and [MS]_1+*m*_TiS_2_ with M = Sn, Pb, or Bi [42,43,64,65,66] (discussed in the previous section) have been studied in depth for their thermoelectric properties. The thermoelectric properties of [MS]_1+*m*_TS_2_ (M = La, Yb; T = Cr, Nb) in the in-plane (*ab*-plane) and out-of-plane (*c*-axis) directions are summarized in Table 2. The NbS_2_ systems show *p*-type *S*, whereas CrS_2_ systems show *n*-type *S*. Among all compounds in the LnS-based series, [Yb_2_S_2_]_0.62_NbS_2_ shows the highest *ZT* of ~0.10 at 300 K because it shows the highest *S*^2^/ρ [81]. The *S* of the Yb sample is ~35%–60% higher than those of samples consisting of the other elements in the lanthanide series at 300 K. This is due to its slightly lower *n* (~5 × 10^20^ cm^−3^), the reason for which is not very well understood and requires further study. At 300 K, a very low κ_lat_ ~0.41 W·K^−1^·m^−1^ is achievable for [Yb_2_S_2_]_0.62_NbS_2_, which reduces even further (~0.18 W·K^−1^·m^−1^) with the removal of ~5% Yb and S from the [Yb_2_S_2_] layer, slightly enhancing the room-temperature *ZT* to ~0.11. This is due to the high phonon scattering originating from the highly modulated structure and atomic deficiency. Further improvements in *ZT* are expected at higher temperatures.

**Table 2:** Seebeck coefficient (*S*); electrical resistivity (ρ); total thermal conductivity (κ_total_); lattice thermal conductivity (κ_lat_); power factor (*S*^2^/ρ); and thermoelectric figure of merit (*ZT*) in the in-plane (*ab*-plane) and out-of-plane (*c*-axis) directions of state-of-the-art misfit layered sulfides: [MS]_1+*m*_TS_2_ (M = La, Yb; T = Cr, Nb) [32,81].

Sample	Direction	*T* (K)	ρ (μΩ·m)	*S* (μV·K^−1^)	κ_total_ (W·K^−1^·m^−1^)	κ_lat_ (W·K^−1^·m^−1^)	*S*^2^/ρ (μW·K^−2^·m^−1^)	*ZT*	Reference
(Yb_2_S_2_)_0.62_NbS_2_	In-plane	300	19.0	60	0.80	0.41	200	0.1	[81]
(La_2_S_2_)_0.62_NbS_2_	In-plane	300	11.5	22	-	-	50	-	[81]
(LaS)_1.14_NbS_2_^a^	In-plane	300	7.6	37	2.50	1.50	177	0.02	[32]
		950	22.0	83	2.00	0.93	316	0.15	
	Out-of-plane	300	13.3	25	2.04	1.48	49	0.01	
		950	32.1	72	1.62	0.88	162	0.09	
(LaS)_1.14_NbS_2_^b^	In-plane	300	5.2	35	4.88	3.45	233	0.02	[32]
		950	16.9	83	3.25	1.86	405	0.15	
	Out-of-plane	300	9.3	25	1.56	0.75	70	0.01	
		950	28.5	56	1.34	0.52	111	0.09	
(LaS)_1.2_CrS_2_^a^	In-plane	950	207	−172	1.16	1.04	143	0.12	[32]
	Out-of-plane	950	223	−174	1.02	0.91	137	0.13	
(LaS)_1.2_CrS_2_^b^	In-plane	950	171	−172	1.25	1.11	174	0.13	[32]
	Out-of-plane	950	278	−154	0.92	0.84	84	0.08	

^a^ Small grains (~1 μm), weak/random orientation of grains; ^b^ Large grains (>20 μm), strong orientation of grains perpendicular to the pressing direction.

As seen from Table 2, misfit compounds are highly sensitive to microstructural variations because of the anisotropic nature of their atomic bonds. Therefore, one strategy to enhance *ZT* is tuning the microstructure. Microstructure control was achieved in (LaS)_1+*m*_TS_2_ (T = Nb, Cr) [32] by varying the sulfurization duration from 6 to 12 h, followed by pressure-assisted sintering to obtain samples with randomly and highly oriented textures. Extended sulfurization (for 12 h) resulted in higher carbon content in the samples, which impeded grain growth [91]. On the other hand, the grains in the sample sulfurized for 6 h grew to >20 μm and self-arranged into a layered structure after sintering (Figure 4b). Large anisotropy was observed in thermoelectric properties for both the systems (Table 2). Both systems show low total thermal conductivity κ_total_, especially in the out-of-plane direction, and the lowest κ_total_ was observed for highly oriented (LaS)_1.2_CrS_2_ (~0.92 W·K^−1^·m^−1^) and (LaS)_1.14_NbS_2_ (~1.34 W·K^−1^·m^−1^), which mainly results from the very low κ_lat_ ~0.84 W·K^−1^·m^−1^ and ~0.52 W·K^−1^·m^−1^, respectively, at 950 K. The low κ_lat_ is attributed to the fact that the interfaces between the host TS_2_ and guest MS layers act as effective phonon scatterers. Similar to ρ and κ_lat_, *S* was found to be highly anisotropic for the highly oriented NbS_2_ misfit compounds. For instance, the in-plane and out-of-plane *S* values were ~83 μV·K^−^^1^ and ~56 μV·K^−^^1^, respectively, for (LaS)_1.14_NbS_2_ at 950 K. The anisotropy in *S* may have originated from the anisotropic band structure. Band-structure calculations showed that strong intralayer energy dispersions (~1.0 eV) occur near the Fermi level, while smaller dispersions of 0.01–0.05 eV occur in the interlayer direction [79,88].

**Figure 8:**
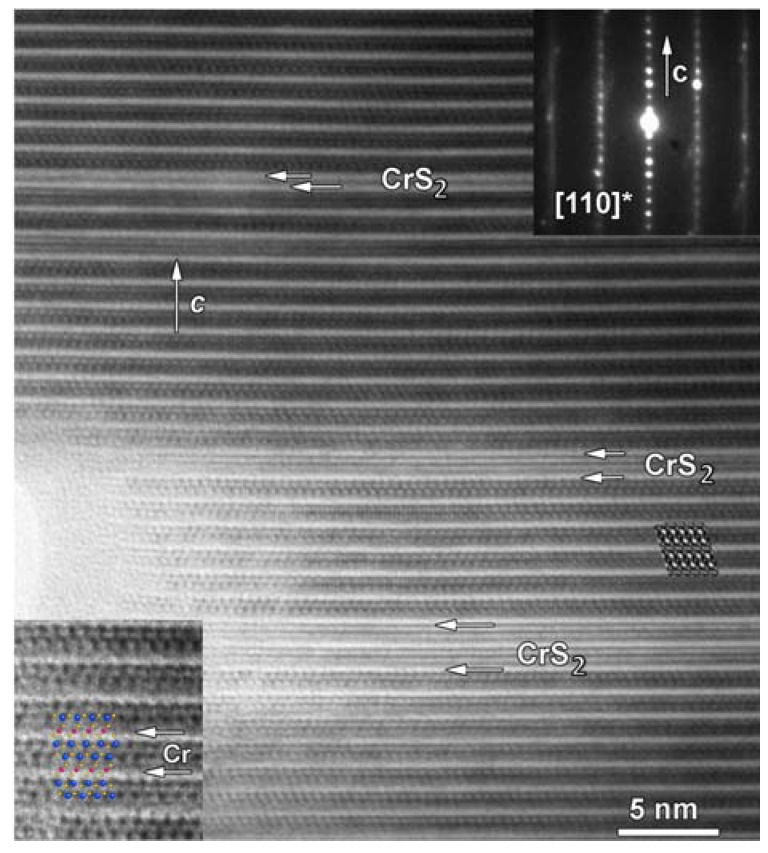
High-resolution transmission electron microscopy (HRTEM) image of (LaS)_1.2_CrS_2_ and corresponding electron diffraction pattern. The structural model superimposed on the enlarged HRTEM image is given as inset in the bottom-left corner of the figure. La, Cr, and S are represented by blue, red, and yellow, respectively. White arrows indicate stacking faults/defects (taken with permission from reference [32]).

Defects such as the presence of extra bright contrast planes are observed in the HRTEM image (indicated by white arrows) and ED patterns (streaks along the *c*-axis) of the CrS_2_ system (Figure 8), which exhibits coherent stacking-fault-induced intergrowth of CrS_2_ layers [32]. With proper manipulation, these crystal defects can help improve the thermoelectric properties. These nanoscale defects, along with the phonon scattering at the atomic scale (layer interfaces, dopants, and point defects) and micro scale (microstructure control) make these compounds good candidates for *ZT* enhancement through all-length-scale hierarchical architecturing [17,18,20].

The highly oriented texture provided the highest *ZT* ~0.14 at 950 K in (LaS)_1.2_CrS_2_, while the sample with weakly oriented texture had the highest *ZT* ~0.15 at 950 K among the (LaS)_1.14_NbS_2_ samples [32]. Carrier mobility (μ) is quite low in these misfit compounds with μ ~ 0.5–1.6 cm^2^·V^−^^1^·s^−^^1^ for the CrS_2_ system and μ ~ 7–11 cm^2^·V^−^^1^·s^−^^1^ for the NbS_2_ system at 300 K. The carrier concentrations at 300 K are *n* ~ 3 × 10^20^ cm^−^^3^ for the CrS_2_ system and *n* ~ 1 × 10^21^ cm^−^^3^ for the NbS_2_ system; these values need to be optimized further for enhancing *S*^2^/ρ. The *ZT* can, therefore, be further improved through appropriate doping and substitutions not only to optimize *n* but also to tune the stacking faults, as reported for the TiS_2_ misfit system [64,66]. These misfit layered sulfides are promising candidates for high-temperature thermoelectric applications as they are environmentally benign, nontoxic, and stable at high temperature; further, they provide tremendous opportunities for enhancing *ZT*.

## 5. Homologous Chalcogenides

**Figure 9:**
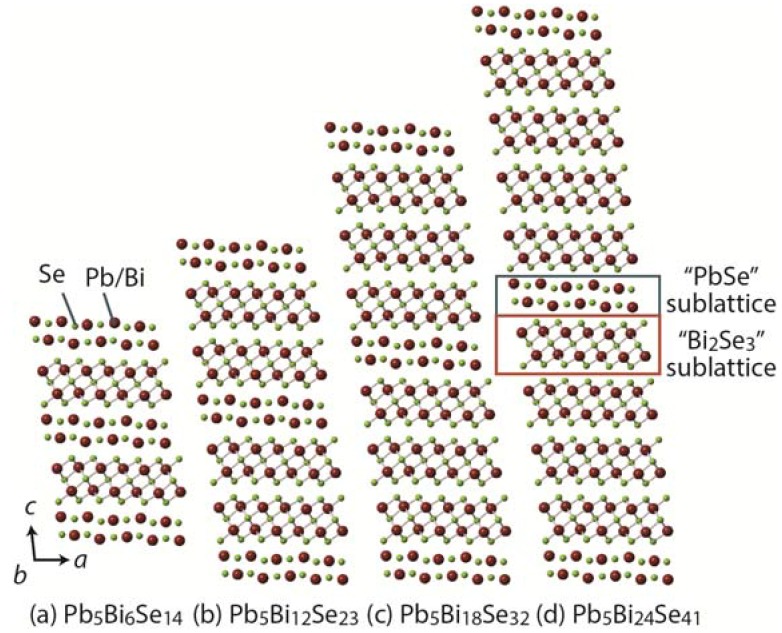
Structural evolution in the cannizzarite homologous series [(PbSe)_5_]*_m_*[(Bi_2_Se_3_)_3_]*_n_*: (**a**) Pb_5_Bi_6_Se_14_ (*m* = 1, *n* = 1); (**b**) Pb_5_Bi_12_Se_23_ (*m* = 1, *n* = 2); (**c**) Pb_5_Bi_18_Se_32_ (*m* = 1, *n* = 3); and (**d**) Pb_5_Bi_24_Se_41_ (*m* = 1, *n* = 4) [33,95,97]. The sizes of atoms in this figure are arbitrary.

A structure of the homologous series is built on the same structural principle with certain module(s) expanding in various dimension(s) by regular increments [92,93,94,95,96]. For example, the crystal structure of the cannizzarite homologous series consists of alternating infinite PbSe- and Bi_2_Se_3_-type layers [33,95,97], as shown in Figure 1c and Figure 9. The two layers are stacked alternately along the *c*-axis, resulting in a three-dimensional structure with varying thicknesses that form different members in the homology. The thermoelectric properties of homologous compounds can be tuned by modifying the size and shape of the structural module(s); therefore, the homologous compounds are good platforms for developing new thermoelectric materials [34,94,98,99,100,101,102,103,104,105,106,107,108,109].

**Figure 10:**
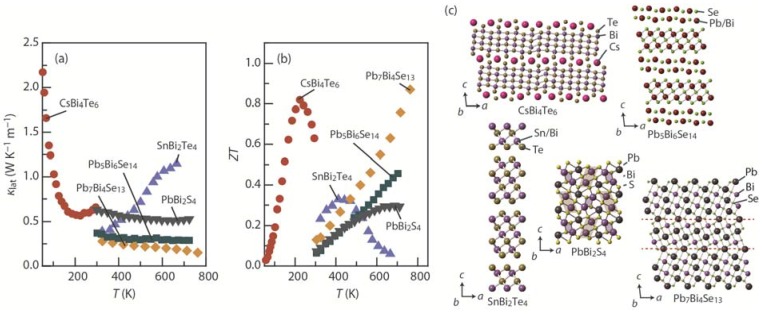
Temperature dependence of the (**a**) lattice thermal conductivity (κ_lat_); (**b**) thermoelectric figure of merit (*ZT*); and (**c**) crystal structure of CsBi_4_Te_6_, a member of the Cs_4_[Bi_2*n*+4_Te_3*n*+6_] homologous series [99,102]; SnBi_2_Te_4_, a member of the [SnTe]*_n_*[Bi_2_Te_3_]*_m_* homologous series [106,107]; Pb_5_Bi_6_Se_14_, a member of the cannizzarite homologous series [34]; PbBi_2_S_4_, a member of the galenobismuthite homologous series [34]; and Pb_7_Bi_4_Se_13_, a member of the lillianite homologous series [109].

Figure 10 shows the κ_lat_, *ZT*, and crystal structure of CsBi_4_Te_6_, a member of the Cs_4_[Bi_2*n*+4_Te_3*n*+6_] homologous series [99,102]; SnBi_2_Te_4_, a member of the [SnTe]*_n_*[Bi_2_Te_3_]*_m_* homologous series [106,107]; Pb_5_Bi_6_Se_14_, a member of the cannizzarite homologous series [34]; PbBi_2_S_4_, a member of the galenobismuthite homologous series [34]; and Pb_7_Bi_4_Se_13_, a member of lillianite homologous series [109]. CsBi_4_Te_6_ and SnBi_2_Te_4_ show *p*-type behavior, while Pb_5_Bi_6_Se_14_, PbBi_2_S_4_, and Pb_7_Bi_4_Se_13_ show *n*-type behavior. The complex crystal structures yield low κ_lat_ in the range of 0.15 to 1.15 W·K^−1^·m^−1^ over the temperature range of 100 K to 763 K, leading to high *ZT*. A record-high *ZT* of ~0.8 at 225 K and a high *ZT* of ~0.9 at 775 K were found in CsBi_4_Te_6_ and Pb_7_Bi_4_Se_13_, respectively.

**Figure 11:**
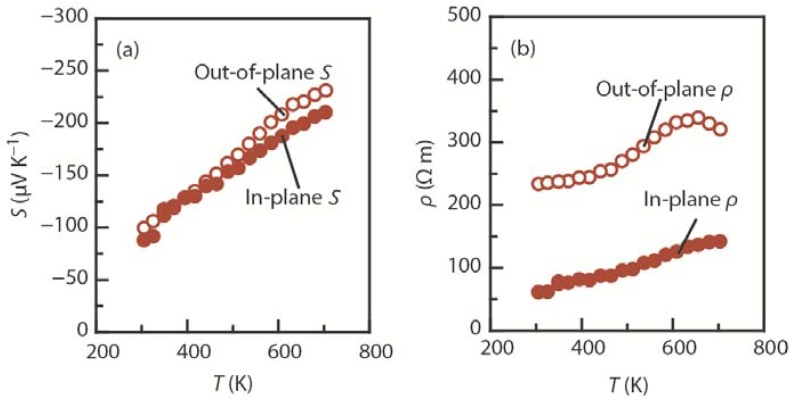

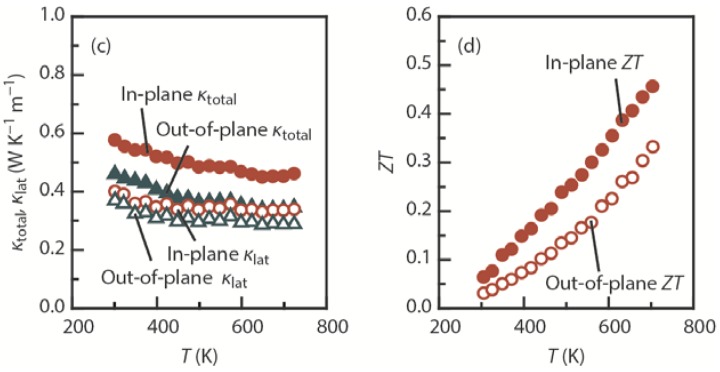
Temperature dependence of the (**a**) Seebeck coefficient (*S*), (**b**) electrical resistivity (ρ), (**c**) total thermal conductivity (κ_total_) and lattice thermal conductivity (κ_lat_), and (**d**) thermoelectric figure of merit (*ZT*) for sintered compacts of the cannizzarite homologous member Pb_5_Bi_6_Se_14_ in the in-plane (*ab*-plane) and out-of-plane (*c*-axis) directions [34].

As shown in Figure 4c, the scanning electron microscope images of the fractured section of sintered compacts of Pb_5_Bi_6_Se_14_ reveal that the crystal grains were preferentially grown in the direction perpendicular to the pressure applied during sintering, forming needle-like grains with a mean length of ~30 μm. The XRD patterns show that the crystalline *c*-axis is preferentially oriented along the pressing (out-of-plane) direction [34]. Figure 11 shows the temperature dependence of the *S*, ρ, κ_total_, κ_lat_, and *ZT* for sintered compacts of the cannizzarite homologous series member Pb_5_Bi_6_Se_14_ in the in-plane and out-of-plane directions. The room-temperature carrier concentration of the system is ~4.8 × 10^19^ cm^−3^. As in TiS_2_, *S* was observed to be insensitive to the crystal orientation (Figure 11a); for example, at 705 K, the in-plane *S* value (~210 μV·K^−1^) is in rough agreement with the out-of-plane *S* value (~230 μV·K^−1^). On the other hand, the interfaces between the PbSe layer and the Bi_2_Se_3_ layer effectively scatter the charge carriers and heat-carrying phonons, leading to highly anisotropic ρ and κ_lat_. For example, at 705 K, the in-plane ρ value (~130 μΩ·m) is 60% lower than the out-of-plane ρ value (~320 μΩ·m) (Figure 11b), and the in-plane κ_lat_ value (~0.39 W·K^−1^·m^−1^) is slightly higher than the out-of-plane κ_lat_ value (~0.31 W·K^−1^·m^−1^) (Figure 11c). The lower ρ in the in-plane direction and the insensitivity of *S* to crystal orientation results in a higher *S*^2^/ρ of 320 μW·K^−2^·m^−1^ and higher *ZT* of 0.46 in the in-plane direction at 705 K (Figure 11d).

One way to enhance the *ZT* of homologous compounds is the tuning of carrier concentration *n* through doping for optimizing *S*^2^/ρ. Another way is to reduce κ_lat_ further by increasing the complexity of the crystal structure using crystal-structural evolution enabled by the homologous series (Figure 9). Table 3 lists the room-temperature thermoelectric properties of three members of the cannizzarite homologous series, Pb_5_Bi_6_Se_14_, Pb_5_Bi_12_Se_23_, and Pb_5_Bi_18_Se_32_ [103]. An important result here is that κ_lat_ systematically reduces from 0.72 W·K^−1^·m^−1^ for Pb_5_Bi_6_Se_14_ (*m* = 1, *n* = 1) to 0.49 W·K^−1^·m^−1^ for Pb_5_Bi_18_Se_32_ (*m* = 1, *n* = 3), demonstrating the reduction in *κ*_lat_ through crystal-structural evolution at the atomic scale.

**Table 3:** Seebeck coefficient (*S*), electrical resistivity (ρ), carrier concentration (*n*), carrier mobility (μ), power factor (*S*^2^/ρ), lattice thermal conductivity (κ_lat_), and thermoelectric figure of merit (*ZT*) at room temperature in three members of the cannizzarite homologous series, Pb_5_Bi_6_Se_14_, Pb_5_Bi_12_Se_23_, and Pb_5_Bi_18_Se_32_ [103].

Sample	*S* (μV·K^−^^1^)	ρ (μΩ·m)	*n* (cm^−^^3^)	μ (cm^2^·V^−^^1^·s^−^^1^)	*S*^2^/ρ (μW·K^−2^·m^−1^)	κ_lat_ (W·K^−1^·m^−1^)	*ZT*
Pb_5_Bi_6_Se_14_	−28	22	0.86 × 10^−^^20^	33	36	0.72	0.01
Pb_5_Bi_12_Se_23_	−27	27	1.15 × 10^−^^20^	20	27	0.59	0.01
Pb_5_Bi_18_Se_32_	−52	39	1.19 × 10^−^^20^	14	69	0.49	0.03

## 6. Accordion-Like Layered SnQ (Q = S, Se)

The major strategies for achieving good thermoelectric performance have mostly involved the use of heavy elements (often toxic elements such as Pb), nanostructuring (which requires precise control of synthesis procedures) [15,16,17,18,19,20,21], and complex unit cells such as skutterudites [22,23,24,25,26,27] and zintl phases [110]. SnSe, however, possesses a very simple crystal structure with light, earth-abundant elements (Figure 1d) and exhibits an intrinsically ultra-low thermal conductivity [35]. SnSe has a layered orthorhombic crystal structure with zigzag (accordion-like) atomic chains (space group *Pnma*) below ~750 K [111]; at ~750 K, it undergoes a phase transition towards its higher-symmetry phase (space group *Cmcm*) [112,113]. SnSe, which has been ignored by the thermoelectric community in the past, has attracted tremendous interest after the very recent report by Kanatzidis *et al.* [35], which demonstrates a record-high *ZT* of ~2.62 at 923 K along the *b*-axis, high *ZT* of ~2.3 along the *c*-axis, and moderate *ZT* of ~0.8 along the *a*-axis in single-crystal SnSe. The lighter analogous sulfide SnS has also received attention in the thermoelectric community [114,115,116].

**Figure 12:**
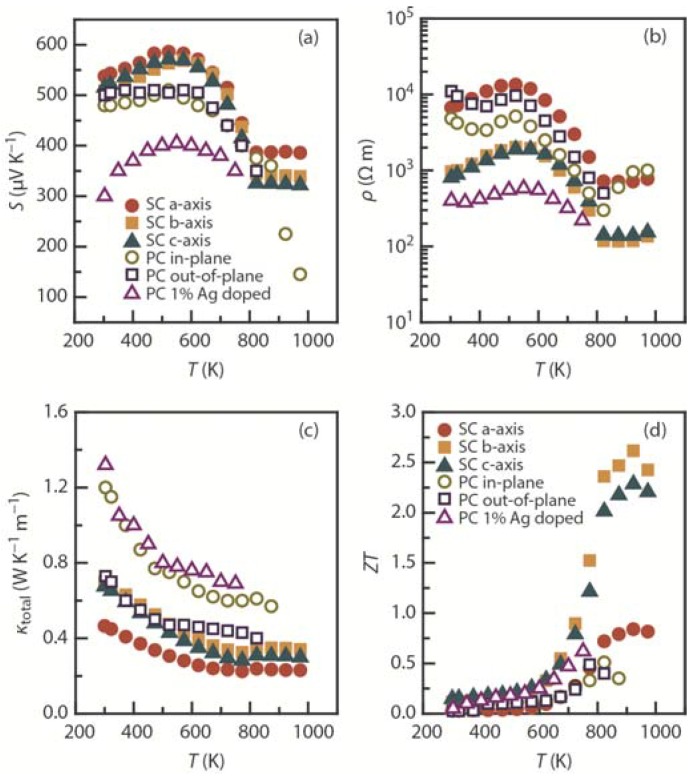
Temperature dependence of the (**a**) Seebeck coefficient (*S*); (**b**) electrical resistivity (ρ); (**c**) total thermal conductivity (κ_total_); and (**d**) thermoelectric figure of merit (*ZT*) for single-crystal (SC) SnSe measured along the *a*-, *b*-, and *c*-axis [35], polycrystalline (PC) SnSe in the in-plane (crystalline *bc*-plane) and out-of-plane (crystalline *a*-axis) directions [117], and 1% Ag-alloyed polycrystalline SnSe in the in-plane direction [118].

Figure 12 shows a comparison of the thermoelectric properties of single-crystal [35], polycrystalline [117], and Ag-alloyed SnSe [118]. There are no significant differences observed *S* between the *a-*, *b-*, and *c*-axis for single-crystal SnSe. *S* decreases at the transition temperature of ~750 K but retains a high value (Figure 12a). For example, the *b*-axis *S* for single crystals decreases from ~570 μV·K^−1^ at 573 K to ~340 μV·K^−1^ at 823 K. Unlike *S*, a large anisotropy in ρ can be seen for the single crystal, where ρ along the *b-* and *c*-axis is much smaller than that along the *a*-axis (Figure 12b). This is because the carrier mobilities μ ~ 250 cm^2^·V^−^^1^·s^−^^1^ in the *b*-axis direction and μ ~ 130 cm^2^·V^−^^1^·s^−^^1^ in the *c*-axis direction at 300 K are higher within the plane of the slab than in the inter-slab direction. The in-plane and out-of-plane ρ of the polycrystalline samples yielded similar observations. The value of ρ dramatically decreases at ~750 K and consequently increases *S*^2^/ρ. In the case of single crystals, the *b*-axis ρ decreases from ~2000 μΩ·m at 573 K to ~120 μΩ·m at 823 K. With Ag alloying of polycrystalline SnSe, the room-temperature carrier concentration increases from 2 × 10^17^ cm^−3^ for a pure system to 9 × 10^18^ cm^−^^3^ for a 7% Ag system [118]. The 1% Ag-alloyed SnSe system produces the highest *ZT* of ~0.6 at 750 K, in contrast to the pure SnSe system, which has a *ZT* of ~0.3 at the same temperature and ~0.5 at 820 K (Figure 12d).

The performance of SnSe surpasses that of other materials, especially above the transition temperature of ~750 K, where the single-crystal κ_lat_ less than 0.25 W·K^−1^·m^−1^ is achieved along all crystallographic axes (Figure 12c) [35]. This value is significantly lower than the κ_lat_ obtained for all-length-scale hierarchically architectured PbTe (PbTe-4 mol% SrTe-2 mol% Na has κ_lat_ ~ 0.5 W·K^−1^·m^−1^ and *ZT* ~ 2.2 at 900 K) [17] and skutterudites exhibiting rattling (for example, LaFe_3_CoSb_12_ has κ_lat_ ~ 1 W·K^−1^·m^−1^ and *ZT* ~ 0.9 at 750 K) [22].

One would expect the κ_total_ (κ_lat_) of polycrystalline samples to be less than that of single-crystal samples because of increased phonon scattering from grain boundaries in polycrystalline samples; however, the opposite is observed for SnSe (Figure 12c). For instance, in-plane κ_total_ values of ~0.6 W·K^−1^·m^−1^ (at 850 K) and ~0.7 W·K^−1^·m^−1^ (at 750 K) were obtained for pure polycrystalline and Ag-alloyed SnSe, respectively. However, very low κ_total_ values of ~0.23 W·K^−1^·m^−1^ (*a*-axis), ~0.34 W·K^−1^·m^−1^ (*b*-axis), and ~0.29 W·K^−1^·m^−1^ (*c*-axis) were obtained for the single-crystal sample at 973 K. *Ab initio* studies suggest a strong anisotropy between all the crystallographic axes with the conductivity in the *b*-axis direction (κ_lat_ ~ 1.4 W·K^−1^·m^−1^) higher than that in the *c*-axis direction (κ_lat_ ~ 0.7 W·K^−1^·m^−1^) at 300 K [119]. This theoretical study relates quite well with the polycrystalline samples but differs markedly from the values for single-crystal samples, in which κ_lat_ values along *b* and *c* axis are reported to be similar (~0.7 W·K^−1^·m^−1^ at 300 K). As data for only two directions (*i.e.*, parallel and perpendicular to the pressing direction) are available for polycrystalline samples, it is difficult to draw conclusions. Furthermore, the room-temperature κ_total_ of the single crystal differs markedly from the earlier reported work on SnSe system [120] and therefore, further verification is warranted. Very recent investigations on Te-substituted SnSe [121] and non-stoichiometric SnSe [118] have indicated that *n*-type conductivity in SnSe is feasible.

The lighter analog SnS is also gaining increased interest, backed up by theoretical work [122,123] supporting the good thermoelectric properties of this compound. SnS also has an accordion-like layered structure and is environmentally compatible and cost-effective. Among the few reports on the thermoelectric properties of SnS [114,115,116], the Ag-doped system demonstrates the highest *ZT* of ~0.6 at 973 K [116]. There is a big scope for improvement in *ZT* through improved doping and an all-length-scale hierarchical architecture approach.

## 7. Thermoelectric Minerals

**Figure 13:**
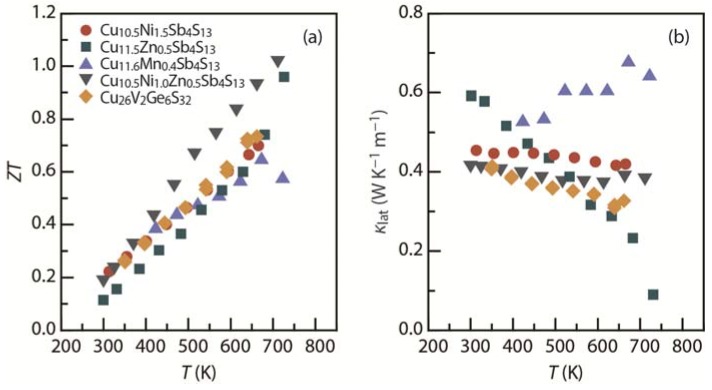
Temperature dependence of (**a**) thermoelectric figure of merit (*ZT*) (*p*-type); and (**b**) lattice thermal conductivity (κ_lat_) for the tetrahedrites Cu_10.5_Ni_1.5_Sb_4_S_13_ [36], Cu_11.5_Zn_0.5_Sb_4_S_13_ [124], Cu_11__.6_Mn_0.4_Sb_4_S_13_ [125], and Cu_10.5_Ni_1.0_Zn_0.5_Sb_4_S_13_ [37] and the colusite Cu_26_V_2_Ge_6_S_32_ [126].

The thermoelectric minerals tetrahedrites (Cu_12_Sb_4_S_13_) and colusites (Cu_26_V_2_M_6_S_32_; M = Ge, Sn) include no layers in their crystal structures. However, the *p*-type *ZT* demonstrated in these mineral-based sulfide systems is among the highest achieved in Pb-free thermoelectric sulfides (Figure 13) and is therefore worth mentioning [36,37,124,125,126].

The terms “tetrahedrite” and “colusite” are derived from naturally occurring minerals (Cu,Fe,Ag,Zn)_12_Sb_4_S_13_ and Cu_24–26_V_2_(As,Sn,Sb)_6_S_32_, respectively, which are mainly composed of the earth-abundant and low-toxicity elements Cu and S. The tetrahedrites possess a cubic structure of $I\bar{4}3m$ symmetry with SbS_3_ pyramids, CuS_4_ tetrahedra, and CuS_3_ triangles. The colusites crystallize in a cubic structure of $P\bar{4}3n$ symmetry with CuS_4_ tetrahedra and VS_4_ tetrahedra.

In 2012, Suekuni *et al.* [127] first reported a notable *S*^2^*/*ρ and low κ_lat_ at room temperature in tetrahedrite systems. Since then, many efforts have been devoted to enhance the *ZT* of tetrahedrite systems [36,37,124,125] and to find new mineral-based thermoelectric materials [126,128]. As shown in Figure 13, mineral-based sulfide systems show extremely low κ_lat_, resulting in high *ZT*. In the case of the tetrahedrites, the low κ_lat_ is due to the low-energy vibration of the Cu atom out of the [CuS_3_] trigonal planar unit, which appears similar to the rattling in skutterudites and clathrates (Figure 1e) [36]. The κ_lat_ of both systems ranges from ~0.1 W·K^−1^·m^−1^ to ~0.7 W·K^−1^·m^−1^ over the temperature range of 300 to 730 K (Figure 13b). The value of *S*^2^/ρ can be increased by tuning the carrier concentration by substituting Tr = Mn [125,127], Fe [124,127], Co [127], Ni [36,37,127], and Zn [37,124,127] at the Cu sites in Cu_12−*x*_Tr*_x_*Sb_4_S_13_. For example, the value of *S*^2^/ρ at 660 K was slightly boosted through Ni doping from 1170 μW·K^−2^·m^−1^ for Cu_12_Sb_4_S_13_ to 1210 μW·K^−2^·m^−1^ for Cu_11.5_Ni_0.5_Sb_4_S_13_. A *ZT* of 1.03 at 723 K was achieved for Cu_10.5_Ni_1.0_Zn_0.5_Sb_4_S_13_ (Figure 13a) [37]. Recently, the structural stability and purity of synthetic Cu_12_Sb_4_S_13_ and Cu_10.4_Ni_1.6_Sb_4_S_13_ were also reported [129]. These high-*ZT* sulfide minerals may help realize feasible methods for environment-friendly and cost-effective thermoelectric waste heat recovery.

## 8. Conclusions and Insights

The decoupling of the interdependent thermoelectric properties of a material has always been a challenge in the development of high-efficiency thermoelectric materials. A layered structure not only provides vast opportunities for enhancing the thermoelectric figure of merit *ZT* through appropriate optimizations at all length scales but also provides methods for individual tuning of thermal and electron transports. Ultra-low lattice thermal conductivities below 0.5 W·K^−1^·m^−1^ have been obtained through intercalation, crystal-structural evolution, or the formation of stacking faults in layered sulfides. Future work must consider scattering centers on all relevant length scales in a hierarchical manner, *i.e.*, from atomic-scale lattice disorders (layer interfaces, substitution sites, and structural evolution) to nano-scale disorders (stacking faults) and microscale grain boundaries (through microtexturing), to achieve the maximum reduction in lattice thermal conductivity. Higher *ZT* is expected with further work to optimize the carrier concentrations in this new category of materials.

## References

[1] Kajikawa T., Rowe D.M. (2005). Thermoelectric power generation system recovering industrial waste heat. Thermoelectrics Handbook: Macro to Nano.

[2] Matsubara K., Matsuura M., Rowe D.M. (2005). A Thermoelectric application to vehicles. Thermoelectrics Handbook: Macro to Nano.

[3] Bell L.E. (2008). Cooling, heating, generating power, and recovering waste heat with thermoelectric systems. Science.

[4] Crane D.T., LaGrandeur J.W. (2010). Progress report on BSST-led US department of energy automotive waste heat recovery program. J. Electron. Mater..

[5] Yang J., Stabler F.R., Rowe D.M. (2012). Automotive applications of thermoelectric materials. Thermoelectrics and Its Energy Harvesting: Modules, Systems, and Applications in Thermoelectrics.

[6] Slack G.A., Rowe D.M. (1995). New materials and performance limits for thermoelectric cooling. CRC Handbook of Thermoelectrics.

[7] Terasaki I., Sasago Y., Uchinokura K. (1997). Large thermoelectric power in NaCo_2_O_4_ single crystals. Phys. Rev. B.

[8] Masset A.C., Michel C., Maignan A., Hervieu M., Toulemonde O., Studer F., Raveau B., Hejtmanek J. (2000). Misfit-layered cobaltite with an anisotropic giant magnetoresistance: Ca_3_Co_4_O_9_. Phys. Rev. B.

[9] Funahashi R., Matsubara I., Ikuta H., Takeuchi T., Mizutani U., Sodeoka S. (2000). An oxide single crystal with high thermoelectric performance in air. Jpn. J. Appl. Phys..

[10] Koumoto K., Terasaki I., Kajitani T., Ohtaki M., Funahashi R., Rowe D.M. (2005). Oxide thermoelectrics. Thermoelectrics Handbook: Macro to Nano.

[11] Koumoto K., Terasaki I., Funahashi R. (2006). Complex oxide materials for potential thermoelectric applications. MRS Bull..

[12] Koumoto K., Wang Y.F., Zhang R.Z., Kosuga A., Funahashi R. (2010). Oxide thermoelectric materials: A nanostructuring approach. Annu. Rev. Mater. Res..

[13] Koumoto K., Funahashi R., Guilmeau E., Miyazaki Y., Weidenkaff A., Wang Y.F., Wan C.L. (2013). Thermoelectric ceramics for energy harvesting. J. Am. Ceram. Soc..

[14] Hébert S., Kobayashi W., Muguerra H., Bréard Y., Raghavendra N., Gascoin F., Guilmeau E., Maignan A. (2013). From oxides to selenides and sulfides: The richness of the CdI_2_ type crystallographic structure for thermoelectric properties. Phys. Status Solidi A.

[15] Biswas K., He J.Q., Zhang Q.C., Wang G.Y., Uher C., Dravid V.P., Kanatzidis M.G. (2011). Strained endotaxial nanostructures with high thermoelectric figure of merit. Nat. Chem..

[16] Ohta M., Biswas K., Lo S.H., He J.Q., Chung D.Y., Dravid V.P., Kanatzidis M.G. (2012). Enhancement of thermoelectric figure of merit by the insertion of MgTe nanostructures in *p*-type PbTe doped with Na_2_Te. Adv. Energy Mater..

[17] Biswas K., He J.Q., Blum I.D., Wu C.I., Hogan T.P., Seidman D.N., Dravid V.P., Kanatzidis M.G. (2012). High-performance bulk thermoelectrics with all-scale hierarchical architectures. Nature.

[18] He J.Q., Kanatzidis M.G., Dravid V.P. (2013). High performance bulk thermoelectrics via a panoscopic approach. Mater. Today.

[19] Zhao L.D., Wu H.J., Hao S.Q., Wu C.I., Zhou X.Y., Biswas K., He J.Q., Hogan T.P., Uher C., Wolverton C. (2013). All-scale hierarchical thermoelectrics: MgTe in PbTe facilitates valence band convergence and suppresses bipolar thermal transport for high performance. Energy Environ. Sci..

[20] Zhao L.D., Dravid V.P., Kanatzidis M.G. (2014). The panoscopic approach to high performance thermoelectrics. Energy Environ. Sci..

[21] Wu H.J., Zhao L.D., Zheng F.S., Wu D., Pei Y.L., Tong X., Kanatzidis M.G., He J.Q. (2014). Broad temperature plateau for thermoelectric figure of merit *ZT* > 2 in phase-separated PbTe_0.7_S_0.3_. Nat. Commun..

[22] Sales B.C., Mandrus D., Williams R.K. (1996). Filled skutterudite antimonides: A new class of thermoelectric materials. Science.

[23] Nolas G.S., Cohn J.L., Slack G.A., Schujman S.B. (1998). Semiconducting Ge clathrates: Promising candidates for thermoelectric applications. Appl. Phys. Lett..

[24] Nolas G.S., Morelli D.T., Tritt T.M. (1999). Skutterudites: A phonon-glass-electron crystal approach to advanced thermoelectric energy conversion applications. Annu. Rev. Mater. Sci..

[25] Guo J.Q., Geng H.Y., Ochi T., Suzuki S., Kikuchi M., Yamaguchi Y., Ito S. (2012). Development of skutterudite thermoelectric materials and modules. J. Electron. Mater..

[26] Takabatake T., Koumoto K., Mori T. (2013). Nano-cage structured materials: Clathrates. Thermoelectric Nanomaterials: Materials Design and Applications.

[27] Rogl G., Grytsiv A., Rogl P., Peranio N., Bauer E., Zehetbauer M., Eibl O. (2014). n-type skutterudites (R,Ba,Yb)*_y_*Co_4_Sb_12_ (R = Sr, La, Mm, DD, SrMm, SrDD) approaching *ZT* ≈ 2.0. Acta Mater..

[28] Salvador J.R., Cho J.Y., Ye Z., Moczygemba J.E., Thompson A.J., Sharp J.W., Koenig J.D., Maloney R., Thompson T., Sakamoto J. (2014). Conversion efficiency of skutterudite-based thermoelectric modules. Phys. Chem. Chem. Phys..

[29] Ohta M., Satoh S., Kuzuya T., Hirai S., Kunii M., Yamamoto A. (2012). Thermoelectric properties of Ti_1+*x*_S_2_ prepared by CS_2_ sulfurization. Acta Mater..

[30] Beaumale M., Barbier T., Bréard Y., Guelou G., Powell A.V., Vaqueiro P., Guilmeau E. (2014). Electron doping and phonon scattering in Ti_1+*x*_S_2_ thermoelectric compounds. Acta Mater..

[31] Meerschaut A., Rabu P., Rouxel J. (1989). Preparation and characterization of new mixed sandwiched layered compounds *Ln*_32_Nb_28_S_88_ (*Ln* = La, Ce). J. Solid State Chem..

[32] Jood P., Ohta M., Nishiate H., Yamamoto A., Lebedev O.I., Berthebaud D., Suekuni K., Kunii M. (2014). Microstructural control and thermoelectric properties of misfit layered sulfides (LaS)_1+*m*_TS_2_ (T = Cr, Nb): The natural superlattice systems. Chem. Mater..

[33] Zhang Y.G., Wilkinson A.P., Lee P.L., Shastri S.D., Shu D., Chung D.Y., Kanatzidis M.G. (2005). Determining metal ion distributions using resonant scattering at very high-energy *K*-edges: Bi/Pb in Pb_5_Bi_6_Se_14_. J. Appl. Crystallogr..

[34] Ohta M., Chung D.Y., Kunii M., Kanatzidis M.G. (2014). Low lattice thermal conductivity in Pb_5_Bi_6_Se_14_, Pb_3_Bi_2_S_6_, and PbBi_2_S_4_: Promising thermoelectric materials in the cannizzarite, lillianite, and galenobismuthite homologous series. J. Mater. Chem. A.

[35] Zhao L.D., Lo S.H., Zhang Y.S., Sun H., Tan G.J., Uher C., Wolverton C., Dravid V.P., Kanatzidis M.G. (2014). Ultralow thermal conductivity and high thermoelectric figure of merit in SnSe crystals. Nature.

[36] Suekuni K., Tsuruta K., Kunii M., Nishiate H., Nishibori E., Maki S., Ohta M., Yamamoto A., Koyano M. (2013). High-performance thermoelectric mineral Cu_12−*x*_Ni*_x_*Sb_4_S_13_ tetrahedrite. J. Appl. Phys..

[37] Lu X., Morelli D.T., Xia Y., Ozolins V. (2015). Increasing the thermoelectric figure of merit of tetrahedrites by Co-doping with nickel and zinc. Chem. Mater..

[38] Rimmington H.P.B., Balchin A.A. (1974). The growth by iodine vapour transport techniques and the crystal structures of layer compounds in the series TiS*_x_*Se_2−*x*_, TiS*_x_*Te_2−*x*_, TiSe*_x_*Te_2−*x*_. J. Cryst. Growth..

[39] Han S.H., Cook B.A. (1995). An experimental search for high ZT semiconductors: A survey of the preparation and properties of several alloy systems. AIP Conf. Proc..

[40] Imai H., Shimakawa Y., Kubo Y. (2001). Large thermoelectric power factor in TiS_2_ crystal with nearly stoichiometric composition. Phys. Rev. B.

[41] Abbott E.E., Kolis J.W., Lowhorn N.D., Sams W., Rao A., Tritt T.M. (2006). Thermoelectric properties of doped titanium disulfides. Appl. Phys. Lett..

[42] Wan C.L., Wang Y.F., Wang N., Koumoto K. (2010). Low-thermal-conductivity (*M*S)_1+*x*_(TiS_2_)_2_ (*M* = Pb, Bi, Sn) misfit layer compounds for bulk thermoelectric materials. Materials.

[43] Wan C.L., Wang Y.F., Wang N., Norimatsu W., Kusunoki M., Koumoto K. (2011). Intercalation: Building a natural superlattice for better thermoelectric performance in layered chalcogenides. J. Electron. Mater..

[44] Guilmeau E., Bréard Y., Maignan A. (2011). Transport and thermoelectric properties in Copper intercalated TiS_2_ chalcogenide. Appl. Phys. Lett..

[45] Gascoin F., Raghavendra N., Guilmeau E., Bréard Y. (2012). CdI_2_ structure type as potential thermoelectric materials: Synthesis and high temperature thermoelectric properties of the solid solution TiS*_x_*Se_2−*x*_. J. Alloy. Compd..

[46] Beaumale M., Barbier T., Bréard Y., Hébert S., Kinemuchi Y., Guilmeau E. (2014). Thermoelectric properties in the series Ti_1−*x*_Ta*_x_*S_2_. J. Appl. Phys..

[47] Beaumale M., Barbier T., Bréard Y., Raveau B., Kinemuchi Y., Funahashi R., Guilmeau E. (2014). Mass fluctuation effect in Ti_1−*x*_Nb*_x_*S_2_ bulk compounds. J. Electron. Mater..

[48] Henderson J.R., Muramoto M., Loh E., Gruber J.B. (1967). Electronic structure of rare-earth sesquisulfide crystals. J. Chem. Phys..

[49] Toide T., Utsunomiya T., Sato M., Hoshino Y., Hatano T., Akimoto Y. (1973). Preparation of lanthanum sulfides using carbon disulfide as sulfurization agent and the change of these sulfides on heating in air. Bull. Tokyo Inst. Technol..

[50] Guittard M., Flahaut J., Meyer G., Morss L.R. (1991). Preparation of rare earth sulfides and selenides. Synthesis of Lanthanide and Actinide Compounds (Topics in f-Element Chemistry, Volume 2).

[51] Hirai S., Shimakage K., Saitou Y., Nishimura T., Uemura Y., Mitomo M., Brewer L. (1998). Synthesis and sintering of cerium(III) sulfide powders. J. Am. Ceram. Soc..

[52] Hirai S., Suzuki K., Shimakage K., Nishimura T., Uemura Y., Mitomo M. (2003). Preparations of γ-Pr_2_S_3_ and γ-Nd_2_S_3_ powders by sulfurization of Pr_6_O_11_ and Nd_2_O_3_ powders using CS_2_ gas, and their sintering. J. Jpn. Inst. Metals.

[53] Miyazaki Y., Ogawa H., Kajitani T. (2004). Preparation and thermoelectric properties of misfit layered sulfide [Yb_1.90_S_2_]_0.62_NbS_2_. Jpn. J. Appl. Phys..

[54] Barin I., Knacke O. (1973). Thermochemical Properties of Inorganic Substances.

[55] Barin I., Knacke O., Kubaschewski O. (1977). Thermochemical Properties of Inorganic Substances: Supplement.

[56] Aoki T., Wan C.L., Ishiguro H., Morimitsu H., Koumoto K. (2011). Evaluation of layered TiS_2_-based thermoelectric elements fabricated by a centrifugal heating technique. J. Ceram. Soc. Jpn..

[57] Li D., Qin X.Y., Zhang J., Wang L., Li H.J. (2005). Enhanced thermoelectric properties of bismuth intercalated compounds Bi*_x_*TiS_2_. Solid State Commun..

[58] Li D., Qin X.Y., Zhang J., Li H.J. (2006). Enhanced thermoelectric properties of neodymium intercalated compounds Nd*_x_*TiS_2_. Phys. Lett. A.

[59] Li D., Qin X.Y., Zhang J. (2006). Improved thermoelectric properties of gadolinium intercalated compounds Gd*_x_*TiS_2_ at the temperatures from 5 to 310 K. J. Mater. Res..

[60] Zhang J., Qin X.Y., Li D., Dong H.Z. (2006). The electrical and thermal conductivity and thermopower of nickel doped compounds (Ni*_x_*Ti_1−*x*_)_1+*y*_S_2_ at low temperatures. J. Phys. D Appl. Phys..

[61] Qin X.Y., Zhang J., Li D., Dong H.Z., Wang L. (2007). The effect of Mg substitution for Ti on transport and thermoelectric properties of TiS_2_. J. Appl. Phys..

[62] Zhang J., Qin X.Y., Li D., Xin H.X., Pan L., Zhang K.X. (2009). The transport and thermoelectric properties of Cd doped compounds (Cd*_x_*Ti_1−*x*_)_1+*y*_S_2_. J. Alloy. Compd..

[63] Zhang J., Qin X.Y., Xin H.X., Li D., Song C.J. (2011). Thermoelectric properties of Co-doped TiS_2_. J. Electron. Mater..

[64] Wan C.L., Wang Y.F., Norimatsu W., Kusunoki M., Koumoto K. (2012). Nanoscale stacking faults induced low thermal conductivity in thermoelectric layered metal sulfides. Appl. Phys. Lett..

[65] Putri Y.E., Wan C.L., Wang Y.F., Norimatsu W., Kusunoki M., Koumoto K. (2012). Effects of alkaline earth doping on the thermoelectric properties of misfit layer sulfides. Scr. Mater..

[66] Putri Y.E., Wan C.L., Dang F., Mori T., Ozawa Y., Norimatsu W., Kusunoki M., Koumoto K. (2014). Effects of transition metal substitution on the thermoelectric properties of metallic (BiS)_1.2_(TiS_2_)_2_ misfit layer sulfide. J. Electron. Mater..

[67] Takeuchi S., Katsuta H. (1970). Characteristics of nonstoichiometry and lattice defects of the TiS_2_ phase. J. Jpn. Inst. Metals.

[68] Thompson A.H., Gamble F.R., Symon C.R. (1975). The verification of the existence of TiS_2_. Mater. Res. Bull..

[69] Murray J.L. (1986). The S-Ti (Sulfur-Titanium) system. J. Phase Equilib..

[70] Kobayashi H., Sakashita K., Sato M., Nozue T., Suzuki T., Kamimura T. (1997). Electronic specific heat of Ti_1+*x*_S_2_ (0 < *x* < 0.1). Physica B.

[71] Cutler M., Leavy J.F., Fitzpatrick R.L. (1964). Electronic transport in semimetallic cerium sulfide. Phys. Rev..

[72] Snyder G.J., Tobere E.S. (2008). Complex thermoelectric materials. Nat. Mater..

[73] May A.F., Fleurial J.-P., Snyder G.J. (2008). Thermoelectric performance of lanthanum telluride produced via mechanical alloying. Phys. Rev. B.

[74] Logothetis E.M., Kaiser W.J., Kukkonen C.A., Faile S.P., Colella R., Gambold J. (1980). Transport properties and the semiconducting nature of TiS_2_. Physica B+C.

[75] Kukkonen C.A., Kaiser W.J., Logothetis E.M., Blumenstock B.J., Schroeder P.A., Faile S.P., Colella R., Gambold J. (1981). Transport and optical properties of Ti_1+*x*_S_2_. Phys. Rev. B.

[76] Koyano M., Negishi H., Ueda Y., Sasaki M., Inoue M. (1986). Electrical resistivity and thermoelectric power of intercalation compounds M*_x_*TiS_2_ (M = Mn, Fe, Co, and Ni). Phys. Status Solidi B.

[77] Amara A., Frongillo Y., Aubin M.J., Jandl S., Lopez-Castillo J.M., Jay-Gerin J.-P. (1987). Thermoelectric power of TiS_2_. Phys. Rev. B.

[78] Wiegers G.A. (1996). Misfit layer compounds: Structures and physical properties. Prog. Solid State Chem..

[79] Van Smaalen S. (1991). Superspace-group approach to the modulated structure of the inorganic misfit layer compound (LaS)_1.14_NbS_2_. J. Phys. Condens. Matter.

[80] Wiegers G.A., Meetsma A., Haange R.J., van Smaalen S., de Boer J.L., Meerschaut A., Rabu P., Rouxel J. (1990). The incommensurate misfit layer structure of (PbS)_1.14_NbS_2_, “PbNbS_3_” and (LaS)_1.14_NbS_2_, “LaNbS_3_”: An X-ray diffraction study. Acta Crystallogr. B.

[81] Miyazaki Y., Ogawa H., Nakajo T., Kikuchii Y., Hayashi K. (2013). Crystal structure and thermoelectric properties of misfit-layered sulfides [Ln_2_S_2_]*_p_*NbS_2_ (Ln = Lanthanides). J. Electron. Mater..

[82] Terashima T., Kojima N. (1994). Electrical transport properties of incommensurate layer compounds (RES)*_x_*NbS_2_ (RE = rare-earth metals; *x* = 1.2, 0.6). J. Phys. Soc. Jpn..

[83] Suzuki K., Enoki T., Imaeda K. (1991). Synthesis, characterization and physical properties of incommensurate layered compounds/(RES)*_x_*TaS_2_ (RE = rare earth metal). Solid State Commun..

[84] Suzuki K., Enoki T., Bandow S. (1993). Electronic properties and valence state of Sm in (SmS)_1.19_TaS_2_. Phys. Rev. B.

[85] Cho N., Kikkawa S., Kanamaru F., Takeda Y., Yamamoto O., Kido H., Hoshikawa T. (1993). Crystal structural, electric and magnetic studies on the misfit layer compounds “LnMS_3_” (Ln = rare-earth metal; M = Ti, V, Cr). Solid State Ion..

[86] Kato K., Kawada I., Takahashi T. (1977). Die Kristallstruktur von LaCrS_3_. Acta Crystallogr. B.

[87] Wiegers G.A., Meerschaut A. (1992). Structures of misfit layer compounds (MS)*_n_*TS_2_ (M = Sn, Pb, Bi, rare earth metals; T = Nb, Ta, Ti, V, Cr; 1.08 < *n* < 1.23). J. Alloy. Compd..

[88] Fang C.M., van Smaalen S., Wiegers G.A., Haas C., de Groot R.A. (1996). Electronic structure of the misfit layer compound (LaS)_1.14_NbS_2_: Band-structure calculations and photoelectron spectra. J. Phys. Condens. Matter.

[89] Fang C.M., Ettema A.R.H., Haas C., Wiegers G.A., van Leuken H., de Groot R.A. (1995). Electronic structure of the misfit-layer compound (SnS)_1.17_NbS_2_ deduced from band-structure calculations and photoelectron spectra. Phys. Rev. B.

[90] Fang C.M., de Groot R.A., Wiegers G.A., Haas C. (1996). Electronic structure of the misfit layer compound (SnS)_1.20_TiS_2_: Band structure calculations and photoelectron spectra. J. Phys. Condens. Matter.

[91] Ohta M., Hirai S., Ma Z., Nishimura T., Uemura Y., Shimakage K. (2006). Phase transformation and microstructures of Ln_2_S_3_ (Ln = La, Sm) with different impurities content of oxygen and carbon. J. Alloy. Compd..

[92] Makovicky E. (1981). The building principles and classification of bismuth-lead sulphosalts and related compounds. Fortschr. Mineral..

[93] Mrotzek A., Kanatzidis M.G. (2003). “Design” in solid-state chemistry based on phase homologies. The concept of structural evolution and the new megaseries A*_m_*[M_1+*l*_Se_2+*l*_]_2*m*_[M_2*l*+*n*_Se_2+3*l*+*n*_]. Acc. Chem. Res..

[94] Kanatzidis M.G., Kanatzidis M.G., Mahanti S.D., Hogan T.P. (2003). New bulk materials for thermoelectric applications: Synthetic strategies based on phase homologies. Chemistry, Physics, and Materials Science of Thermoelectric Materials.

[95] Kanatzidis M.G. (2005). Structural evolution and phase homologies for “design” and prediction of solid-state compounds. Acc. Chem. Res..

[96] Makovicky E. (2006). Crystal structures of sulfides and other chalcogenides. Rev. Mineral. Geochem..

[97] Segawa K., Taskin A.A., Ando Y. (2015). Pb_5_Bi_24_Se_41_: A new member of the homologous series forming topological insulator heterostructures. J. Solid State Chem..

[98] Chung D.-Y., Choi K.-S., Iordanidis L., Schindler J.L., Brazis P.W., Kannewurf C.R., Chen B., Hu S., Uher C., Kanatzidis M.G. (1997). High thermopower and low thermal conductivity in semiconducting ternary K-Bi-Se compounds. Synthesis and properties of *β*-K_2_Bi_8_Se_13_ and K_2.5_Bi_8.5_Se_14_ and their Sb analogues. Chem. Mater..

[99] Chung D.Y., Hogan T., Brazis P., Rocci-Lane M., Kannewurf C., Bastea M., Uher C., Kanatzidis M.G. (2000). CsBi_4_Te_6_: A high-performance thermoelectric material for low-temperature applications. Science.

[100] Kuznetsova L.A., Kuznetsov V.L., Rowe D.M. (2000). Thermoelectric properties and crystal structure of ternary compounds in the Ge(Sn,Pb)Te–Bi_2_Te_3_ systems. J. Phys. Chem. Solids.

[101] Caillat T., Huang C.K., Fleurial J.-P., Snyder G.J., Borshchevsky A., Rowe D.M. (2000). Synthesis and thermoelectric properties of some materials with the PbBi_4_Te_7_ crystal structure. Proceedings of the 19th International Conference on Thermoelectrics.

[102] Chung D.Y., Hogan T.P., Rocci-Lane M., Brazis P., Ireland J.R., Kannewurf C.R., Bastea M., Uher C., Kanatzidis M.G. (2004). A new thermoelectric material: CsBi_4_Te_6_. J. Am. Chem. Soc..

[103] Shelimova L.E., Karpinskii O.G., Konstantinov P.P., Avilov E.S., Kretova M.A., Lubman G.U., Nikhezina I.Yu., Zemskov V.S. (2010). Composition and properties of compounds in the PbSe-Bi_2_Se_3_ system. Inorg. Mater..

[104] Zemskov V.S., Shelimova L.E., Konstantinov P.P., Avilov E.S., Kretova M.A., Nikhezina I.Y. (2011). Thermoelectric materials with low heat conductivity based on PbSe-Bi_2_Se_3_ compounds. Inorg. Mater. Appl. Res..

[105] Zemskov V.S., Shelimova L.E., Konstantinov P.P., Avilov E.S., Kretova M.A., Nikhezina I.Yu. (2012). Thermoelectric materials based on layered chalcogenides of bismuth and lead. Inorg. Mater. Appl. Res..

[106] Kuropatwa B.A., Kleinke H. (2012). Thermoelectric properties of stoichiometric compounds in the (SnTe)*_x_*(Bi_2_Te_3_)*_y_* system. Z. Anorg. Allg. Chem..

[107] Kuropatwa B.A., Assoud A., Kleinke H. (2013). Effects of cation site substitutions on the thermoelectric performance of layered SnBi_2_Te_4_ utilizing the triel elements Ga, In, and Tl. Z. Anorg. Allg. Chem..

[108] Medlin D.L., Snyder G.J. (2013). Atomic-scale interfacial structure in rock salt and tetradymite chalcogenide thermoelectric materials. JOM.

[109] Olvera A., Shi G.G., Djieutedjeu H., Page A., Uher C., Kioupakis E., Poudeu P.F.P. (2015). Pb_7_Bi_4_Se_13_: A lillianite homologue with promising thermoelectric properties. Inorg. Chem..

[110] Kauzlarich S.M., Brown S.R., Snyder G.J. (2007). Zintl phases for thermoelectric devices. Dalton Trans..

[111] Lefebvre I., Szymanski M.A., Olivier-Fourcade J., Jumas J.C. (1998). Electronic structure of tin monochalcogenides from SnO to SnTe. Phys. Rev. B.

[112] Chattopadhyay T., Pannetier J., von Schnering H.G. (1986). Neutron diffraction study of the structural phase transition in SnS and SnSe. J. Phys. Chem. Solids.

[113] Peters M.J., McNeil L.E. (1990). High-pressure Mössbauer study of SnSe. Phys. Rev. B.

[114] Fadavieslam M.R., Shahtahmasebi N., Rezaee-Roknabadi M., Bagheri-Mohagheghi M.M. (2011). A study of the photoconductivity and thermoelectric properties of Sn*_x_*S*_y_* optical semiconductor thin films deposited by the spray pyrolysis technique. Phys. Scr..

[115] Tan Q., Li J.F. (2014). Thermoelectric properties of Sn-S bulk materials prepared by mechanical alloying and spark plasma sintering. J. Electron. Mater..

[116] Tan Q., Zhao L.D., Li J.F., Wu C.F., Wei T.R., Xing Z.B., Kanatzidis M.G. (2014). Thermoelectrics with earth abundant elements: Low thermal conductivity and high thermopower in doped SnS. J. Mater. Chem. A.

[117] Sassi S., Candolfi C., Vaney J.-B., Ohorodniichuk V., Masschelein P., Dauscher A., Lenoir B. (2014). Assessment of the thermoelectric performance of polycrystalline p-type SnSe. Appl. Phys. Lett..

[118] Chen C.L., Wang H., Chen Y.Y., Day T., Snyder G.J. (2014). Thermoelectric properties of *p*-type polycrystalline SnSe doped with Ag. J. Mater. Chem. A.

[119] Carrete J., Mingo N., Curtarolo S. (2014). Low thermal conductivity and triaxial phononic anisotropy of SnSe. Appl. Phys. Lett..

[120] Wasscher J.D., Albers W., Haas C. (1963). Simple evaluation of the maximum thermoelectric figure of merit, with application to mixed crystals SnS_1-*x*_Se*_x_*. Solid-State Electron..

[121] Chen S., Cai K., Zhao W. (2012). The effect of Te doping on the electronic structure and thermoelectric properties of SnSe. Physica B.

[122] Parker D., Singh D.J. (2010). First Principles investigations of the thermoelectric behavior of tin sulfide. J. Appl. Phys..

[123] Bera C., Jacob S., Opahle I., Gunda N.S.H., Chmielowski R., Dennler G., Madsen G.K.H. (2014). Integrated computational materials discovery of silver doped tin sulfide as a thermoelectric material. Phys. Chem. Chem. Phys..

[124] Lu X., Morelli D.T., Xia Y., Zhou F., Ozolins V., Chi H., Zhou X.Y., Uher C. (2013). High performance thermoelectricity in earth-abundant compounds based on natural mineral tetrahedrites. Adv. Energy Mater..

[125] Chetty R., Kumar D.S.P., Rogl G., Rogl P., Bauer E., Michor H., Suwas S., Puchegger S., Giester G., Mallik R.C. (2015). Thermoelectric properties of a Mn substituted synthetic tetrahedrite. Phys. Chem. Chem. Phys..

[126] Suekuni K., Kim F.S., Nishiate H., Ohta M., Tanaka H.I., Takabatake T. (2014). High-performance thermoelectric minerals: Colusites Cu_26_V_2_M_6_S_32_ (M = Ge, Sn). Appl. Phys. Lett..

[127] Suekuni K., Tsuruta K., Ariga T., Koyano M. (2012). Thermoelectric properties of mineral tetrahedrites Cu_10_*Tr*_2_Sb_4_S_13_ with low thermal conductivity. Appl. Phys. Express.

[128] Tsujii N., Mori T. (2014). Stability and thermoelectric property of Cu_9_Fe_9_S_16_: Sulfide mineral as a promising thermoelectric material. MRS Proc..

[129] Barbier T., Lemoine P., Gascoin S., Lebedev O.I., Kaltzoglou A., Vaqueiro P., Powell A.V., Smith R.I., Guilmeau E. (2015). Structural stability of the synthetic thermoelectric ternary and nickel-substituted tetrahedrite phases. J. Alloy. Compd..

