# Correction: Jood, P. and Ohta, M. Hierarchical Architecturing for Layered Thermoelectric Sulfides and Chalcogenides. *Materials* 2015, *8*, 1124–1149

**DOI:** 10.3390/ma8095315

**Published:** 2015-09-21

**Authors:** Priyanka Jood, Michihiro Ohta

**Affiliations:** Energy Technology Research Institute, National Institute of Advanced Industrial Science and Technology (AIST), Tsukuba, Ibaraki 305-8568, Japan; E-Mail: p.jood@aist.go.jp

The authors wish to make the following corrections to this paper [1].

The authors regret that the lattice thermal conductivity (*κ*_at_) values of some samples in Table 1 and thermoelectric figure of merit (*ZT*) values of some samples in Table 2 were not correct. The tables with correct *κ*_lat_ and *ZT* values are shown below. The authors would like to apologize for any inconvenience caused.

**Table 1:** Seebeck coefficient (*S*), electrical resistivity (ρ), carrier mobility (μ), power factor (*S*^2^/ρ), lattice thermal conductivity (*κ*_lat_), and thermoelectric figure of merit (*ZT*) at room temperature in the in-plane (*ab*-plane) and out-of-plane (*c*-axis) directions for a single crystal of nearly stoichiometric TiS_2_ [2] and polycrystalline Ti_1.008_S_2_ [3].

Sample	Direction	*S* (μV·K^−1^)	ρ (μΩ m)	μ (cm^2^·V^−1^·s^−1^)	*S*^2^/ρ (μW·K^−2^·m^−1^)	*κ*_lat_ (W·K^−1^·m^−1^)	*ZT*
Single crystal	In-plane	−251	17	15	3710	6.35	0.16
Single crystal	Out-of-plane	-	13,000	0.017	-	4.21	-
Polycrystalline	In-plane	−80	6.2	2.3	1030	2.0	0.12
Polycrystalline	Out-of-plane	−84	11	1.2	630	1.8	0.10

**Table 2:** Seebeck coefficient (*S*), electrical resistivity (ρ), total thermal conductivity (κ_total_), lattice thermal conductivity (κ_lat_), power factor (*S*^2^/ρ), and thermoelectric figure of merit (*ZT*) in the in-plane (*ab*-plane) and out-of-plane (*c*-axis) directions of state-of-the-art misfit layered sulfides: [MS]_1+*m*_TS_2_ (M = La, Yb; T = Cr, Nb) [4,5].

Sample	Direction	*T* (K)	ρ (μΩ·m)	*S* (μV·K^−1^)	κ_total_ (W·K^−1^·m^−1^)	κ_lat_(W·K^−1^·m^−1^)	*S*^2^/ρ (μW·K^−2^·m^−1^)	*ZT*	Reference
(Yb_2_S_2_)_0.62_NbS_2_	In-plane	300	19.0	60	0.80	0.41	200	0.1	[5]
(La_2_S_2_)_0.62_NbS_2_	In-plane	300	11.5	22	-	-	50	-	[5]
(LaS)_1.14_NbS_2_^a^	In-plane	300	7.6	37	2.5	1.50	177	0.02	[4]
		950	22.0	83	2.00	0.93	316	0.15	
	Out-of-plane	300	13.3	25	2.04	1.48	49	0.01	
		950	32.1	72	1.62	0.88	162	0.09	
(LaS)_1.14_NbS_2_^b^	In-plane	300	5.2	35	4.88	3.45	233	0.02	[4]
		950	16.9	83	3.25	1.86	405	0.12	
	Out-of-plane	300	9.3	25	1.56	0.75	70	0.01	
		950	28.5	56	1.34	0.52	111	0.08	
(LaS)_1.2_CrS_2_^a^	In-plane	950	207	−172	1.16	1.04	143	0.11	[4]
	Out-of-plane	950	223	−174	1.02	0.91	137	0.13	
(LaS)_1.2_CrS_2_^b^	In-plane	950	171	−172	1.25	1.11	174	0.14	[4]
	Out-of-plane	950	278	−154	0.92	0.84	84	0.08	

^a^ Small grains (~1 μm), weak/random orientation of grains; ^b^ Large grains (>20 μm), strong orientation of grains perpendicular to the pressing direction.

## References

[1] Jood P., Ohta M. (2015). Hierarchical architecturing for layered thermoelectric sulfides and chalcogenides. Materials.

[2] Imai H., Shimakawa Y., Kubo Y. (2001). Large thermoelectric power factor in TiS2 crystal with nearly stoichiometric composition. Phys. Rev. B.

[3] Ohta M., Satoh S., Kuzuya T., Hirai S., Kunii M., Yamamoto A. (2012). Thermoelectric properties of Ti1+*x*S2 prepared by CS2 sulfurization. Acta Mater..

[4] Jood P., Ohta M., Nishiate H., Yamamoto A., Lebedev O.I., Berthebaud D., Suekuni K., Kunii M. (2014). Microstructural control and thermoelectric properties of misfit layered sulfides (LaS)1+mTS2 (T = Cr, Nb): The natural superlattice systems. Chem. Mater..

[5] Miyazaki Y., Ogawa H., Nakajo T., Kikuchii Y., Hayashi K. (2013). Crystal structure and thermoelectric properties of misfit-layered sulfides [Ln2S2]*p*NbS2 (Ln = Lanthanides). J. Electron. Mater..

